# Fireworks ocular injury in Saudi children: profile and management outcomes

**DOI:** 10.1038/s41598-022-09606-x

**Published:** 2022-04-08

**Authors:** Huda AlGhadeer, Rajiv Khandekar

**Affiliations:** 1 Emergency Department, King Khaled Eye Specialist Hospital, PO Box 7191, Riyadh, 11462 Kingdom of Saudi Arabia; 2 Research Department, King Khaled Eye Specialist Hospital, Riyadh, Kingdom of Saudi Arabia; 3 British Columbia Center for Epidemiologic and International Ophthalmology, UBC, Vancouver, Canada

**Keywords:** Diseases, Health care

## Abstract

To explore the demographic profiling, causes, types, complications, management outcomes, and severity of fireworks-inflicted ocular injuries in children in KSA. This is a retrospective study of 115 cases with eye injuries managed at the Emergency Department, of our institution between 2003 and 2019. Demography, clinical features at presentation, mode of management and the Best-corrected visual acuity (BCVA) were evaluated at the last follow up. The study included 117 eyes of 115 children [median age: 9 years; 96 (83.5%) boys;19 (16.5%) girls]. Fifty-six (48.7%) participants were bystanders. The injuries were caused mainly due to bangers (n = 47; 40.9%), rockets in bottle (n = 28; 24.3%), firecrackers (n = 27; 23.5%), and nonspecific reasons (n = 13; 11.3%). The children had presented with various severity levels: corneal abrasion (n = 52; 44.4%); cataract (n = 47;40.2%); penetrating injury (n = 40; 34.2%); secondary glaucoma (n = 22;18.8%); subluxated lens (n = 19;16.2%); limbal stem cell deficiency (n = 14;12.0%); Iridodialysis (n = 12;10.3%), and vitreous hemorrhage (n = 11;9.4%). Management interventions of the eyes under study included: penetrating injury repair (n = 40; 34.2%), lens removal plus intraocular lens implantation (n = 26; 22.2%), removal of foreign body (n = 9; 7.7%). The BCVA after six months was 20/20 to 20/60 in 49 (41%) cases; 20/70 to 20/200 in 27 (23.1%) cases; < 20/200 to 20/400 in 7 (6%) cases, and < 20/400 in 34 (29.1%) of the cases. Out of 51.3% eyes with < 20/200 before management, only 35% recorded severe visual impairment. Fireworks-related eye injuries were mainly observed in boys primarily due to the use of bangers. Visual disability remained in one-third of the managed cases.

## Introduction

Ocular injuries often lead to acquired monocular blindness in children^1^. Firework is employed to express rejoicement on the eve of New Year and several National Days^2,3^ in various countries. Several studies have reported damage in an average of 21.8% of^4^ individuals: ranging from 16 to 45% in Europe^4^. The USA records about 31% of fireworks-related ocular injuries during Independence Day celebrations on 4th July^5^ every year. Wisse et al.^4^ documented that in both developing and developed nations, 22% of all ocular trauma result from fireworks, thereby becoming the driving reason for permanent and/or partial visual disability^2,4^. 47% of ocular patients were reported to result from 7000 fireworks-related injuries treated in the USA in one month^6^.

Unprofessional and novice handling of fireworks may inflict severe forms of trauma and may even lead to death if not timely and professionally treated^7,8^. Explosive physical and chemical reactions triggered during fireworks explosions may injure eyes and hands most and fatally affect these organs^3^. The Public Prosecution Law and its strict implementation in KSA have warned of serious implications due to fireworks misuse and mishandling. It stipulates that the authorized person is a natural or legal person who meets the necessary conditions and is authorized by the Ministry of Interior (MOI), KSA. Manufacture, possession, export, import, sell, use, distribution, transportation, and storage of fireworks stand banned in KSA unless authorized by the MOI in accordance with the enabling legal provisions. The Emergency Department (ED) of our institution is a leading ophthalmic specialized center. All patients with isolated eye injuries and different body injuries are treated in a general hospital. Ocular injuries caused by fireworks^2–9^ were reported in several previous studies. However, little data about the epidemiology of fireworks-related ocular injuries among children and information on the type of fireworks in Saudi Arabia are recorded in literature. To our knowledge, the current study is the first such study in the Kingdom of Saudi Arabia that delved deeply into fireworks-related ocular conditions in children.

The authors in this study worked assiduously to highlight clinical features, profiling, associated outcomes, management patterns of ocular trauma in children recruited due to fireworks-related injuries at an emergency department (ED) of a tertiary eye hospital from 2003 through 2019. This paper also elaborates on preventative methods and regulations to further mitigate ocular fireworks-related injuries.

## Materials and methods

The current retrospective study was conducted at the Emergency department (ED) of our institution, Riyadh, KSA, over 16 years (2003–2019). After taking approval from the Institutional Review Board of the hospital, medical records of all children under 16 years who presented to the (ED) with ocular injuries from fireworks were evaluated. Ophthalmologists in the emergency unit, having rich experience in the patient assessment and management of ocular trauma and its complications, conducted the study.

The demographic profile of patients, including their age, gender, laterality (right, left, or bilateral/both eyes), types of injuries sustained, and nature of fireworks employed, were recorded. The status of the children as active participants or bystanders was also ascertained. A proper regulatory written consent from the parents of children admitted to the hospital was obtained to publish images of injuries and treatments; management patterns such as types and number of operations, duration from injury to admission.

Visual Acuity at presentation and after management; anterior and posterior segment findings; and surgical interventions were noted. A detailed ophthalmological examination was performed on all patients at the time of presentation and on follow-up, best-corrected visual acuity (BCVA) was recorded at 6 m with the participant wearing the best correction if warranted. Intraocular pressure (Goldmann applanation or rebound tonometry using ICare device in children and required palpation) was assessed. Pupillary examination and inspection of the adnexa were done; the anterior ocular segment was evaluated using slit-lamp biomicroscopy, and loupes in combination with a light source in uncooperative children were employed. Indirect ophthalmoscopy was employed to study injury in the ocular fundus. In the case of obstructed or invisible/inconspicuous image of fundus, B-scan ultrasonography was performed. In suspected cases of penetrating injury, B-scan ultrasonography was exempted.

During the investigations, an urgent computed tomography (CT) scan was ordered in suspected intraocular foreign bodies (IOFB) and suspected orbital trauma including bony fractures. The children were admitted to the hospital for more delicate or extensive surgery, especially those done under general anesthesia, and intraocular surgery for intravenous drug application or an intensive eye drop regimen (e.g., in alkali burns). Intravenous antibiotics were provided to children presenting with open-globe trauma to tackle endophthalmitis and in cases of extensive eyelid or orbital injuries. Tetanus vaccination was also given. Necessary surgical interventions were conducted within 24 h of admission to the (ED). All patients were classified based on the ocular trauma classification system^10^. Lid repair, Lens removal plus intraocular lens implantation, removal of foreign body¸ amniotic membrane graft transplantation, pars plana vitrectomy, and enucleation were recorded.

Uncorrected visual acuity (UCVA) and best-corrected visual acuity (BCVA) were tested through Snellen charts, Cardiff visual acuity cards, or finger counting at presentation and after management. Poor visual outcome was defined as BCVA < 6/60 and unilateral blindness as BCVA < 3/60 in the injured eye. The visual impairment grades defined by the World Health Organization^11^ were adopted in the current study.

Data were collected using Excel (Microsoft Office 2010, Redmond, Seattle, USA). Univariate analysis was performed using SPSS, version 25.0 (IBM Corp., Armonk, NY, USA). Normally distributed quantitative variables were expressed both as the mean and standard deviation (SD). Qualitative variables were presented as frequencies and percentage proportions. For subgroup analysis validation of continuous variables, a Student t-test was performed. A two-sided P-value was calculated with P < 0.05 considered as statistically significant.

### Statement of ethics

The local ethics committee of the King Khaled Eye Specialist Hospital approved the protocol, and it adhered to the tenets of the Declaration of Helsinki. The nature of the study and its possible consequences were explained to study participants. Informed consent was obtained from a parent/or legal guardian to participate in this study in addition to consent to publish images.

## Results

One hundred-seventeen eyes of 115 children with a median age of 9 (25% IQR: 6, range 2–14) years were investigated and treated in the current study. A higher ocular injury rate (83.5%) was recorded in 96 boys than in girls (19, 16.5%). The right eye was involved in 56 (48.7%) patients and the left eye in 57 (49.6%) patients. In only two patients (1.7%), both eyes were seen injured Table 1.

**Table 1:** Profile of Saudi children with fireworks-related ocular injuries.

Qualitative variables	Number	Percentage
**Age group (years)**		
< 5	19	16.5
5 to 9.9	51	44.4
10 to 14.9	45	39.1
**Gender**		
Boys	96	83.5
Girls	19	16.5
**Eye involved**		
Right	56	48.7
Left	57	49.6
Both	2	1.7
**Bystander/igniting fireworks**		
Bystander	56	48.7
Igniting fireworks	59	51.3
**Type of fireworks**		
Banger	47	40.9
Rockets in bottle	28	24.3
Firecrackers	27	23.5
Unknown	13	11.3

**Figure 1:**
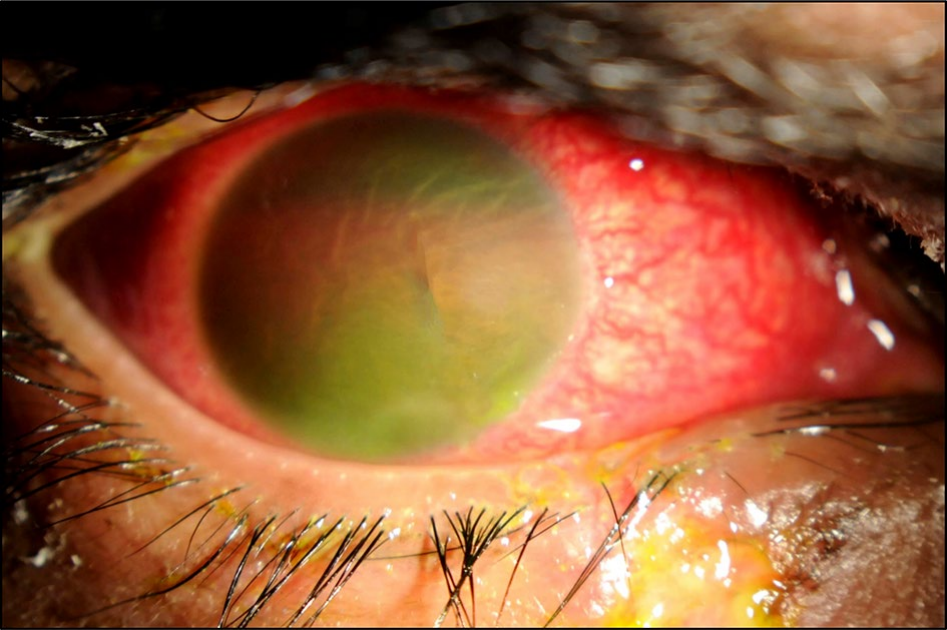
Slit-lamp photo showing hyphema caused by fireworks injury.

Fifty-six (48.7%) patients were bystanders, and 59 (51.3%) had ignited the fireworks themselves while sustaining ocular injuries. Types of fireworks used by the injured patients were banger in 47 (40.9%); rockets in bottle 28 (24.3%); firecrackers in 27 (23.5%); and unspecified in 13 (11.3%) patients. There is no significant difference in the visual outcome by type of causative agent. Table 2. Out of 115 patients, 44.4% had corneal abrasion, 40.2% had cataract, 34.2% had Penetrating injury, 16.2% had the subluxated lens, 10.3% had iridodialysis, 8.5% had hyphema (Fig. 1) and 9.4% had a vitreous hemorrhage Table 3.

**Table 2:** Visual outcomes (BCVA) by the type of fireworks-related ocular injury.

BCVA	Firecrackers		Rockets in bottle		Banger		Unknown		Validation
	Number	Percentage	Number	Percentage	Number	Percentage	Number	Percentage	
20/20 to 20/60	9	33.3	11	39.3	23	48.9	5	38.5	Chi square = 7.4; Df = 9; P = 0.6
20/60 to 20/200	10	37	7	25	6	12.8	4	30.8	
20/200 to 20/400	1	3.7	3	10.7	3	6.4	1	7.7	
Less than 20/400	7	26	7	25	15	31.9	3	23	
Total	27	100	28	100	47	100	13	100	

*BCVA* best corrected visual acuity.

**Table 3:** Ocular profile of Saudi children with fireworks-related injuries.

N = 117 eyes of 115 children	Number	Percentage
**Presented best corrected visual acuity BCVA**		
20/20 to 20/60	26	22.2
20/70 to 20/200	31	26.5
< 20/200 to 20/400	3	2.6
< 20/400	57	48.7
**Damage in the anterior segment of the eye**		
Conjunctival wound/burn	5	4.3
Corneal abrasion	52	44.4
Corneal foreign bodies	7	6.0
Penetrating injury	40	34.2
Hyphema	10	8.5
Iritis	11	9.4
Iridodialysis	12	10.3
Cataract	47	40.2
Subluxated lens	19	16.2
Others/unspecified	23	19.7
**Damage in the posterior segment of the eye**		
Vitreous hemorrhage	11	9.4
Retinal detachment	6	5.1
Choroidal detachment	1	0.9
IOFB	9	7.7
Optic nerve damage	1	0.9
Extrusion of eyeball contents	1	0.9
Commotio retinae	1	0.9

*BCVA* best corrected visual acuity, *IOFB* intraocular foreign bodies.

**Figure 2:**
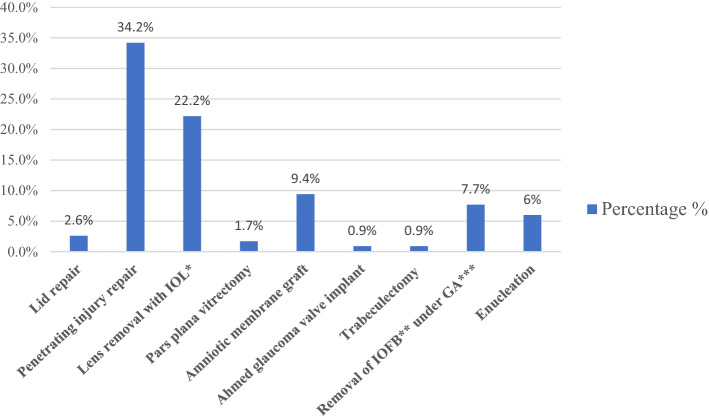
Ocular surgeries performed on the eyes of Saudi children with fireworks-related injuries. Abbreviations: *IOL Intraocular lens, **IOFB Intraocular foreign bodies, ***GA General anesthesia.

Only 100 children underwent an operation. Management included penetrating injury repair (34.2%), lens removal plus intraocular lens (IOL) implantation (22.2%), removal of intraocular foreign bodies (IOFB) (7.7%), amniotic membrane graft transplantation (9.4%), and other surgeries (3.3%). Enucleation was felt necessary in seven children (Fig. 2).

Table 4 reveals visual outcomes (BCVA) by the type of the first surgery performed to manage fireworks-related injury. The variation in visual impairment grade in the injured eyes by type of management was significant. Late complications in the eyes of Saudi children following fireworks-related injuries are displayed in Table 5.

**Table 4:** Visual outcomes stratified by the type of the first surgery performed to manage fireworks-related ocular injury in Saudi children.

BCVA	Penetrating injury repair		Lens removal + (IOL) Implantation		Others		Validation
	Number	Percentage	Number	Percentage	Number	Percentage	
20/20 to 20/60	12	30	11	42.3	10	29.4	Chi square = 6.4; Df = 6; P = 0.238
20/60 to 20/200	20	50	7	26.9	14	41.2	
20/200 to 20/400	0	0.0	2	7.7	3	8.8	
Less than 20/400	8	20	6	23.1	7	20.6	
Total	40	100	26	100	34	100	

*BCVA* best corrected visual acuity, *IOL* intraocular lens implantation.

**Table 5:** Late complications in the eyes of Saudi children following fireworks-related ocular injuries.

Late complications	Frequency (n)	Percentage (%)
Corneal opacity	25	21.3
Secondary Glaucoma	22	18.8
Traumatic cataract	21	17.9
Limba stem cell deficiency	14	12
Angle recession	13	11
Recurrent Retinal detachment	6	5
Traumatic macular hole	4	3.4
Phthisis bulbi	2	1.7

Secondary procedures included lens removal with IOL implantation, amniotic membrane transplantation, enucleation, and retinal surgeries for recurrent retinal detachment. Six patients underwent ruptured globe repair and retinal surgeries for recurrent retinal detachment and proliferative vitreoretinopathy, and four for traumatic macular holes were performed at 3–6 months of follow-up. Two patients sustained a devastating ruptured globe needing primary enucleation, and 5 needed subsequent enucleation. Twenty-one underwent lens removal with IOL implantation. Thirteen children had angle recession, and 22 who developed secondary glaucoma are on regular follow-up. After trabeculectomy failed to control intraocular pressure, the Ahmed glaucoma valve implant was used as a second-line operation to manage intraocular pressure in the same patient. On a follow-up of 1–3 years, corneal scarring was noted in 21% of cases, and keratoplasty with Amniotic membrane transplantation was needed in 12 children. In thirteen cases, follow-up data were not available.

Fifteen children needed no surgeries and were managed in the outpatient department. The average follow-up period after the presentation was six months and ranged from 1 to 3 years from surgical management.

In the 117 eyes, the initial and BCVA, at last, follow up are shown in Table 6. The BCVA after six months was 20/20 to 20/60 in 49 (41%) eyes; 20/70 to 20/200 in 27 (23.1%); and < 20/200 to 20/400 in 7 (6%) and < 20/400 in (34;29.1%) eyes, from 51.3% eyes with < 20/200 before management, we could reduce severe visual impairment to 35.1%.

**Table 6:** Visual status before and after the management of Saudi children with fireworks-related ocular injuries.

BCVA	After management		Before management		Validation
	Eyes	Percentage	Eyes	Percentage	
20/20 to 20/60	49	41.9	26	22.2	χ^2^ = 11, Degree of Freedom = 3; P < 0.001
20/70 to 20/200	27	23.1	31	26.5	
< 20/200 to 20/400	7	6.0	3	2.6	
< 20/400	34	29	57	48.7	

*BCVA* best-corrected visual acuity.

## Discussion

The findings indicated that young male children are predominantly affected by unilateral trauma. Bangers followed by rockets in bottle were the main types of fireworks causing ocular injuries. Corneal abrasions, ruptured globes, and cataracts were the principal ocular diagnosis. The chief cause triggering ocular injuries was the ignition of the fireworks. Nearly half of the affected individuals displayed normal functional vision six months after treatment management. Prompt and standard intervention could reduce blindness by one-third.

Several studies were conducted previously on ocular injuries in children and on outcomes of standard management from other parts of the globe, except on an Arab population. With strict laws preventing fireworks in KSA, availability and unsupervised usage are concerned.

Ocular trauma is a crucial reason for causing monocular visual morbidity and blindness^12,13^. Several studies have outlined fireworks-related ocular injuries sustained during ceremonies in different countries^8,14–17^ Al-Qattan and Al-Tamimi in their study on hand burns due to fireworks^18^ in KSA indicated that wherever firework-related injuries were noted, ocular injuries could also be present and needed an evaluation.

In the current study, half of the children were under 10 years of age. This indicates the vulnerability of children to ocular trauma due to fireworks. This finding was also corroborated by a few studies done in India^13,19^. In the USA 10- to 20-year-old children comprised one-third of injured children^20^. This is attributed to lack of supervision, little experience handling fireworks, more risk-taking behavior, and lower ability to respond to dangerous hazards. These observations highlight the need for greater education on the dangers that fireworks pose and implementation at earlier school age.

This study also showed that the majorities of the fireworks (40.9%) were bangers, followed by rockets in bottle (24.3%) and firecrackers (23.5%). In 13 eyes (11.3%), unknown fireworks were employed. In an earlier study, firecrackers were shown to be the most used type of fireworks^14^. Use of bottle rockets was not remarkable. Sparklers, another type of fireworks, were found associated with corneal abrasions and burns. The 1999 US Consumer Product Safety Commission (CPSC) study documented that one-third of the fireworks-related injuries were caused by firecrackers and 20% by rockets^9^.

Severe trauma was reported in 34% of the patients under study, which indicated that about one-third of fireworks-related ocular injuries could cause permanent sequelae. A significant proportion of patients (85%) required surgical interventions. The most frequently performed surgeries were ruptured globe repair, cataract extraction, amniotic membrane graft, and removal of intraocular foreign bodies (IOFB), which reflect the severity of these injuries. The ocular injuries varied in presentation and severity. Injuries led to a 29% resulted in permanent eyesight loss, as evidenced by the mean Snellen visual acuity of < 20/400. Previous studies^5,15^ also found that open globe injury, poor initial visual acuity, IOFB, and retinal detachment were also associated with poor visual outcomes.

Our finding revealed an enucleation rate of 6%. This finding concurs with the 3.9% enucleation rate documented by Wisse et al.^4^ in their review of the literature covering 40 years span (1969–2009). This observation highlights the need to save eyes and employ ultra-advanced treatment options in the current dynamic world. Chang et al.^5^ reported an enucleation rate of 10% between 2003 and 2013 and more open-globe injuries (17%) vs. 6% in the current study. This is likely due to the fact, that Chang et al.^5^ studied the experience of the sole level I trauma center for five U.S. states.

In our study, nearly half of the injured eyes had normal functional vision after the intervention. One-third of eyes developed unilateral blindness after the intervention compared to nearly half at presentation. Overall, children in the current study exhibited enormous improvement in their visual acuity post-treatment. However, a remarkable number of injuries triggered permanent vision loss in the patients. A better recovery to normal vision could be attributed to the absence of retinal detachment and IOFB, better initial BCVA, and closed globe injury. Visual acuity witnessed noteworthy improvement on account of prompt interventions.

Open globe injury and IOFB were found associated with dismally poor visual outcomes. Poor visual outcomes of interventions following fireworks-inflicted ocular injuries^13,15,17^ might arise due to risk factors such as IOFB, retinal detachment, open globe injury, poor initial visual acuity, and development of endophthalmitis. Patients with open globe injuries and retained IOFB warrant a diligent prognosis.

Boys of 6 to 10 years of age in our study seemed to be more vulnerable to fireworks-related ocular injuries. This matches the findings of Malik et al.^15^ who reported that 54% of boys with fireworks-related injuries were ≤ 14 years of age. Our study indicates that boys are the major victims of eye injuries in 83.5% of cases caused by fireworks because they are actively involved in lighting fireworks; this is consistent with findings from previous studies^2,5,19–24^, probably reflecting the males to be more adventurous and hostile. We also observed that the active participants were 51.3%, while the bystanders were 48.7% of patients. This finding contradicts findings from previous studies^15,24^. It can be concluded that active participants are more easily injured than bystanders.

The most common injuries were corneal abrasions, ruptured globes, and cataracts. Ruptured globes occurred in 34% of patients, similar to previously reported rates^3^. We found corneal abrasions in about 44% of patients, showing conformity with finding by Wisse et al.^4^.

This study has several limitations. It is a retrospective study. All data could not be retrieved from the children’s medical records. Data concerning visual acuity at presentation were especially lacking. Furthermore, children were unable to identify their injuries. Due to the long distance from the hospital, many patients could not be followed-up and were lost to follow-up.

Fireworks-related injuries are preventable to a large extent. Studies showed that that countries with stricter laws had 87% fewer ocular injuries than those with relaxed rules about the private use of fireworks^25^. Children are more likely than adults to sustain fireworks-related eye injuries^26^. Strict regulations concerning the use of fireworks are needed for a significant protective impact on the children since minors sustain a profound proportion of fireworks-inflicted severe injuries^26^. Minors are undoubtedly placed at a greater risk for severe ocular trauma. The Saudi Arabian laws regulate the free distribution and ban the use of explosives for private fireworks. Pediatricians need to create awareness among parents, community leaders, children, etc. about the dangers involved in fireworks. Public sales of all fireworks should be prohibited. International trade in fireworks for private use needs to be prohibited. Spectators need to keep them away from the area where the fireworks are ignited. In case of eye injury, the eye should not be touched, or no attempt should be made to treat the wound, and emergency medical help should be sought forthwith without brooking any delay. Parents should educate minors, particularly adolescents, about the proper use of fireworks to prevent the burgeoning rates of injuries in minors.

## Conclusion

Fireworks-related ocular injuries could have serious repercussions for patients with ocular morbidity and visual acuity, particularly in severe trauma affecting younger patients. This study presents the most comprehensive data on the outcomes of fireworks-related ocular damage in children in KSA. In our study, nearly half of the affected eyes had normal functional vision after the intervention, and one-third of the eyes had unilateral blindness, compared to almost half at the presentation. However, a significant proportion of injuries resulted in children losing their vision permanently. Fireworks, which are believed to represent happiness, offer an inconceivable risk to the eyes of innocent users. The current study indicated that additional policies regarding fireworks use are required to reduce the incidence of visual morbidity in children.

## Abbreviations

BCVA: Best corrected visual acuity
USA: United States of America
KSA: Kingdom of Saudi Arabia
ED: Emergency Department
CT: Computed tomography
SPSS: Statistical Package for Social Sciences
IOL: Intraocular lens
IOFB: Intraocular foreign body

## References

[1] Puodžiuvienė E, Jokūbauskienė G, Vieversytė M (2018). A five-year retrospective study of the epidemiological characteristics and visual outcomes of pediatric ocular trauma. BMC Ophthalmol..

[2] Frimmel S, Theusinger OM, Kniestedt C (2017). Analysis of ocular firework-related injuries and common eye traumata: A 5-year clinical study. Klin. Monbl. Augenheilkd..

[3] Lenglinger MA, Zorn M, Pilger D (2021). Firework-inflicted ocular trauma in children and adults in an urban German setting. Eur. J. Ophthalmol..

[4] Wisse RP, Bijlsma WR, Stilma JS (2010). Ocular firework trauma: A systematic review on incidence, severity, outcome, and prevention. Br. J. Ophthalmol..

[5] Chang IT, Prendes MA, Tarbet KJ (2016). Ocular injuries from fireworks: The 11-year experience of a US level I trauma center. Eye.

[6] Tu YL, Granados DV (2014). Fireworks-related deaths, emergency department-treated injuries, and enforcement activities during. US Consumer Product Safety Commission Report..

[7] Wang C, Zhao R, Du WL (2014). Firework injuries at a major trauma and burn center: A five-year prospective study. Burns.

[8] Frimmel S, de Faber JT, Wubbels RJ (2018). Type, severity, management and outcome of ocular and adnexal firework-related injuries: The Rotterdam experience. Acta Ophthalmol..

[9] Billock RM, Chounthirath T, Smith GA (2017). Pediatric firework-related injuries presenting to United States Emergency Departments, 1990–2014. Clin. Pediatr..

[10] Pieramici DJ, Sternberg P, Aaberg TM (1997). A system for classifying mechanical injuries of the eye (globe). Am. J. Ophthalmol..

[11] World Health Organization. *International Statistical Classification of Diseases and Related Health Problems: Alphabetical Index*. World Health Organization; 2004.

[12] Myers J, Lehna C (2017). Effect of fireworks laws on pediatric fireworks-related burn injuries. J. Burn Care Res..

[13] John D, Philip SS, Mittal R (2015). Spectrum of ocular firework injuries in children: A 5-year retrospective study during a festive season in Southern India. Indian J. Ophthalmol..

[14] Sandvall BK, Jacobson L, Miller EA (2017). Fireworks type, injury pattern, and permanent impairment following severe fireworks-related injuries. Am. J. Emerg. Med..

[15] Malik A, Bhala S, Arya SK (2013). Five-year study of ocular injuries due to fireworks in India. Int. Ophthalmol..

[16] Rashid RA, Heidary F, Hussein A (2011). Ocular burns and related injuries due to fireworks during the Aidil Fitri celebration on the East Coast of the Peninsular Malaysia. Burns.

[17] Shiuey EJ, Kolomeyer AM, Kolomeyer NN (2020). Assessment of firework-related ocular injury in the US. JAMA Ophthalmol..

[18] Al-Qattan MM, Al-Tamimi AS (2009). Localized hand burns with or without concurrent blast injuries from fireworks. Burns.

[19] Venkatesh R, Gurav P, Tibrewal S (2017). Appraising the spectrum of firework trauma and the related laws during Diwali in North India. Indian J. Ophthalmol..

[20] Moore JX, McGwin G, Griffin RL (2014). The epidemiology of firework-related injuries in the United States: 2000–2010. Injury.

[21] Qi Y, Zhu Y (2013). Prognostic value of an ocular trauma score in ocular firecracker trauma. J. Burn Care Res..

[22] Bagri N, Saha A, Chandelia S (2013). Fireworks injuries in children: A prospective study during the festival of lights. Emerg. Med. Australas..

[23] Jing Y, Yi-Qiao X, Yan-Ning Y (2010). Clinical analysis of firework-related ocular injuries during Spring Festival 2009. Graefes Arch. Clin. Exp. Ophthalmol..

[24] Rishi E, Rishi P, Koundanya VV (2016). Post-traumatic endophthalmitis in 143 eyes of children and adolescents from India. Eye.

[25] Jeyabal P, Davies L, Rousselot A (2019). Fireworks: boon or bane to our eyes?. International ophthalmology..

[26] AlGhadeer H, Khandekar R (2021). Profile and management outcomes of fireworks-related eye injuries in Saudi Arabia: A 16-year retrospective study. Clin Ophthalmol..

